# Novel Rechargeable M_3_V_2_(PO_4_)_3_//Zinc (M = Li, Na) Hybrid Aqueous Batteries with Excellent Cycling Performance

**DOI:** 10.1038/srep25809

**Published:** 2016-05-12

**Authors:** H. B. Zhao, C. J. Hu, H. W. Cheng, J. H. Fang, Y. P. Xie, W. Y. Fang, T. N. L. Doan, T. K. A. Hoang, J. Q. Xu, P. Chen

**Affiliations:** 1 College of Science, Shanghai University, Shanghai 200444, China; 2 Department of Chemical Engineering, University of Waterloo, Waterloo N2L 5G5, Canada; 3 School of Materials Science and Engineering, Shanghai University, Shanghai 200444, China; 4 State Key Laboratory of Transducer Technology, Shanghai Institute of Microsystem and Information Technology, Chinese Academy of Sciences, Shanghai 200050, People’s Republic of China

## Abstract

A rechargeable hybrid aqueous battery (ReHAB) containing NASICON-type M_3_V_2_(PO_4_)_3_ (M = Li, Na) as the cathodes and Zinc metal as the anode, working in Li_2_SO_4_-ZnSO_4_ aqueous electrolyte, has been studied. Both of Li_3_V_2_(PO_4_)_3_ and Na_3_V_2_(PO_4_)_3_ cathodes can be reversibly charge/discharge with the initial discharge capacity of 128 mAh g^−1^ and 96 mAh g^−1^ at 0.2C, respectively, with high up to 84% of capacity retention ratio after 200 cycles. The electrochemical assisted ex-XRD confirm that Li_3_V_2_(PO_4_)_3_ and Na_3_V_2_(PO_4_)_3_ are relative stable in aqueous electrolyte, and Na_3_V_2_(PO_4_)_3_ showed more complicated electrochemical mechanism due to the co-insertion of Li^+^ and Na^+^. The effect of pH of aqueous electrolyte and the dendrite of Zn on the cycling performance of as designed MVP/Zn ReHABs were investigated, and weak acidic aqueous electrolyte with pH around 4.0–4.5 was optimized. The float current test confirmed that the designed batteries are stable in aqueous electrolytes. The MVP//Zn ReHABs could be a potential candidate for future rechargeable aqueous battery due to their high safety, fast dynamic speed and adaptable electrochemical window. Moreover, this hybrid battery broadens the scope of battery material research from single-ion-involving to double-ions -involving rechargeable batteries.

Energy storage and transition systems, including Li//air^1^,^2^, Mg/air^3^, metal (V, Sn, Ni)//air^4^, Zn//air^5^, Tin/Air^6^, aqueous rechargeable lithium/sodium batteries^1^,^7^,^8^,^9^,^10^, redox flow batteries^11^, Li//I_2_^12^, Li//sulphur^13^, and Li//Se^14^, Fuel cell^15^,^16^, capacitor^17^,^18^, have been developed rapidly to satisfy the various energy demands of electronics and electric automobiles. Recently, based on aqueous rechargeable battery -rechargeable hybrid aqueous batteries (ReHABs)- have been widely developed^6^,^7^,^8^,^9^ on account of their safety, environmental friendliness, and low cost. ReHAB is indeed a novel power system, mainly because of its separated electrochemical mechanism in cathode and anode. In most aqueous rechargeable batteries, only one type ion (Li^+^) involved in the electrochemical reactions traveling back and forth between electrodes, such as the well-known “rocking chair” mechanism in lithium-ion batteries. In ReHABs, lithium ions are intercalated/extraction in the composite cathode (second-order electrode), while zinc ions are deposited/dissolved from the zinc metal anode (first-order electrode). This novel battery broadens the scope of battery research from single-ion-involving to double-ions-involving rechargeable batteries.

Zinc is abundant, low cost and environmental friendly in the nature with a high theoretical capacity (820 mAhg^−1^) as anode. An adaptable negative potential (−0.762 V vs. SHE)^19^, enable it to be constructed with other cathodes in aqueous electrolytes. Several rechargeable zinc batteries, including nickel//zinc battery^20^, zinc//air battery^21^ and Zn//Na_0.95_MnO_2_^7^, Zn//LiMn_2_O_4_^8^,^22^ have been investigated in aqueous systems.

However, there are not many choices of cathodes using in ReHABs for the limitation of their potentials vs. Zn^2+^/Zn. Contradictorily, too low cathode potential also reduces the power density of cathodes, while too high potential will destroy the aqueous electrolyte, as well as decomposition of water. Totally, the cathodes which can be used in aqueous electrolyte required aqueous stability, adaptable charge/discharge potential plateaus compared with that of decomposition of water. Although the 4 V materials such as LiMn_2_O_4_^23^,^24^, LiCoO_2_^25^ or LiNi_1/3_Co_1/3_Mn_1/3_O_2_^26^ were introduced into aqueous electrolyte batteries, their potential plateaus in aqueous media are close to water decomposition potential. Therefore, those cathodes with potential plateaus below 4 V show more promising in aqueous electrolyte batteries. Only few cathodes are more possible in Li-containing ReHABs with possible good cycling performance in aqueous electrolytes, including Li_3_V_2_(PO_4_)_3_ (LVP)^27^,^28^,^29^,^30^, Na_3_V_2_(PO_4_)_3_ (NVP)^31^,^32^,^33^,^34^, LiFePO_4_^10^, and MnO_2_^35^,^36^.

The monoclinic Li_3_V_2_(PO_4_)_3_ and rhombohedral Na_3_V_2_(PO_4_)_3_ have attracted considerable interest as promising cathode candidate in non-aqueous lithium-ion batteries due to their thermodynamically stable structure, high cell-voltage and fairish theoretical specific capacity (LVP: 191 mAh g^−1^ for extraction of three Li^+^ ions between 3.0–4.6 V, 133 mAh g^−1^ for extraction of two Li^+^ ions between 3.0–4.2 V vs. Li^+^/Li; NVP: theoretical discharge capacity with 118 mAhg^−1^, with two Na^+^ ions being completely extracted up to 4.0 V versus Na^+^/Na).^19^,^20^,^21^,^22^,^23^,^24^. The (PO_4_)^3−^ polyanions facilitate to form a three dimensional pathway for Li^+^ insertion /extraction with a very high Li ion diffusion coefficient (from 10^−9^ to 10^−10^ cm^2^/s). As cathodes of hybrid ion batteries, Li_2_NaV_2_(PO_4_)_3_(LNVP) were also investigated in organic lithium ion electrolytes^37^,^38^. These two phosphate salts, LVP and NVP, have obvious different insertion/extraction mechanisms in organic electrolyte, including the inserted/extracted lithium ion number per unit formula and plateaus voltages as well structure stability, but their behaviour in aqueous electrolyte seldom is evaluated. In this work, we studied these cathodes to expand their application in aqueous electrolyte batteries.

By calculation, the discharge plateau voltages (vs. Zn^2+^/Zn) of LVP should be at 1.25 V, 1.35 V, 1.75 V and 2.2 V. Detailedly, the plateau at 1.25 V and 1.35 V is correspond to the insertion of one lithium ion, and the plateau at 1.75 V is for the insertion of the second lithium ion, which are lower than the oxygen evolution reaction (OER) of water. The plateau voltage around 2.2 V is higher than that of OER, therefore the third lithium of LVP is impossible to be extracted in aqueous electrolyte. The theoretical energy density of LVP//Zn and NVP//Zn batteries calculated by the total weight of cathodes and Zn anode is about 164 Wh kg^−1^ and 76 Wh kg^−1^, respectively. Therefore, the electrolyte stability in ReHABs with LVP cathode should be higher than that of ReHABs containing LiMn_2_O_4_ (2.05 V vs. Zn^2+^/Zn), considering that the working potential of LiMn_2_O_4_ is closer to the OER of aqueous electrolyte. LVP also has higher charge/discharge plateaus than that of LiFePO_4_(1.45 V vs. Zn^2+^/Zn), therefore, the power density should be optimism. The discharge plateau voltages (vs. Zn^2+^/Zn) of NVP should be at 1.35 V, and one Na^+^ ion can be extracted. The fast ion transfer and reversible charge/discharge performance of MVP in organic encourage us to study its applications in aqueous electrolytes. Like most cathodes in organic or aqueous electrolyte, MVP cathodes also face some challenges, such as structure stable ability and cycling performance when they are introduced into a new system, which attract our interest to study.

Inspired by this fact, we developed a new binary ion-containing ReHAB with M_3_V_2_(PO_4_)_3_ (MVP, M = Li, Na) and zinc as cathode and anode respectively, operated in an aqueous electrolyte containing Zn^2+^ and Li^+^ (Na^+^) (Scheme 1). Their electrochemical performance and dynamic reaction mechanism are investigated. As well, the main effect of pH and concentration of electrolyte, float charge current and Zn dendrite on the electrochemical performance were evaluated.

## Result and Discussion

**Figure 1:**
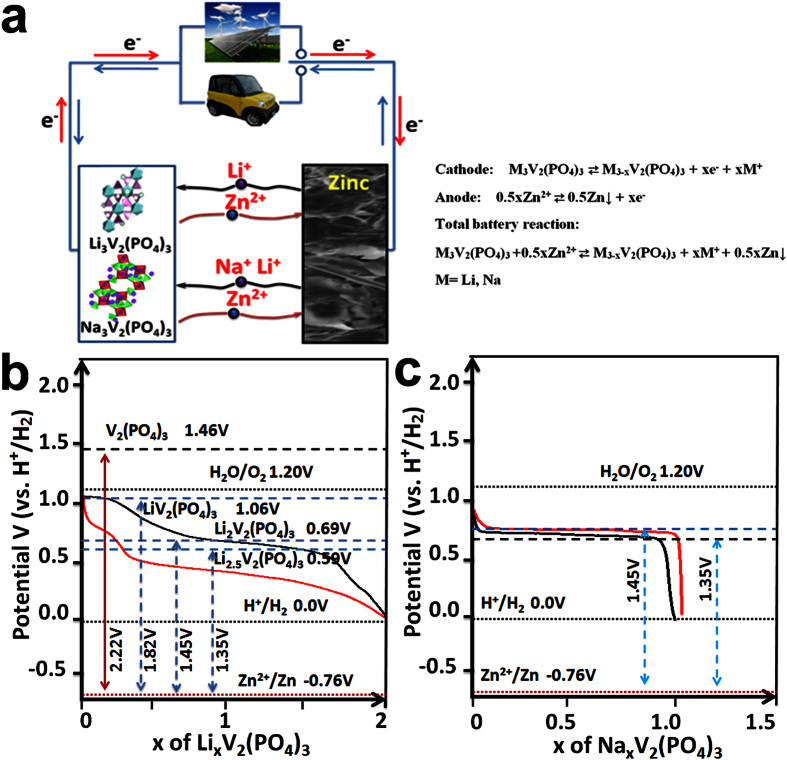
The operation scheme of MVP//Zn ReHABs (**a**); The charge/discharge plateaus potentials of LVP (**b**) and NVP (**c**) cathodes with Zn as counter electrode working in hybrid aqueous electrolyte with current density of 0.2C.

In this work, we designed a two-ions (Zn^2+^ and Li^+^) involving aqueous electrolyte rechargeable battery with MVP (M = Li, Na) as cathode and Zn foil as anode, respectively (Fig. 1a). According to the charge/discharge profile of LVP in organic electrolyte with Li metal as anode we reported^39^,^40^, there should be at least four pairs of charge/discharge plateaus on LVP cathode in the potential window from 0 to 1.75 V vs. H^+^/H_2_. However, in the real LVP/Zn (Fig. 1b) battery with 1M Li_2_SO_4_ + 2M ZnSO_4_ (pH = 3.7) as electrolyte, the charge/discharge profile shows that this ReHABs can reversibly work with capacity of 120 mAhg^−1^ and there are three pairs of charge/discharge plateaus (1.45/1.50 V, 1.85/2.05 V, 1.35/1.50 V) in a voltage range of 0.7 V to 2.1 V vs. Zn^2+^/Zn. The fourth plateaus at higher potential (>2.2 V) could not be observed because of serious decomposition of hydrogen oxide. As for the NVP cathode, the charge/discharge plateaus in organic electrolyte shows that only one sodium ion can be extracted and lithium/sodium ion co-insertion occurred in discharge process with one pair of plateaus around 3.75 V/3.65 V^37^,^40^. In this work, NVP//Zn ReHABs is predicted reversely charging/discharging, and two pairs of plateaus with one lithium ion extraction per formula at 1.35 V and 1.45 V vs Zn^2+^/Zn were observed in Fig. 1c. In short, these two MVP//Zn ReHABs show promising application on energy storage with excellent safety and high specific capacity.

**Figure 2:**
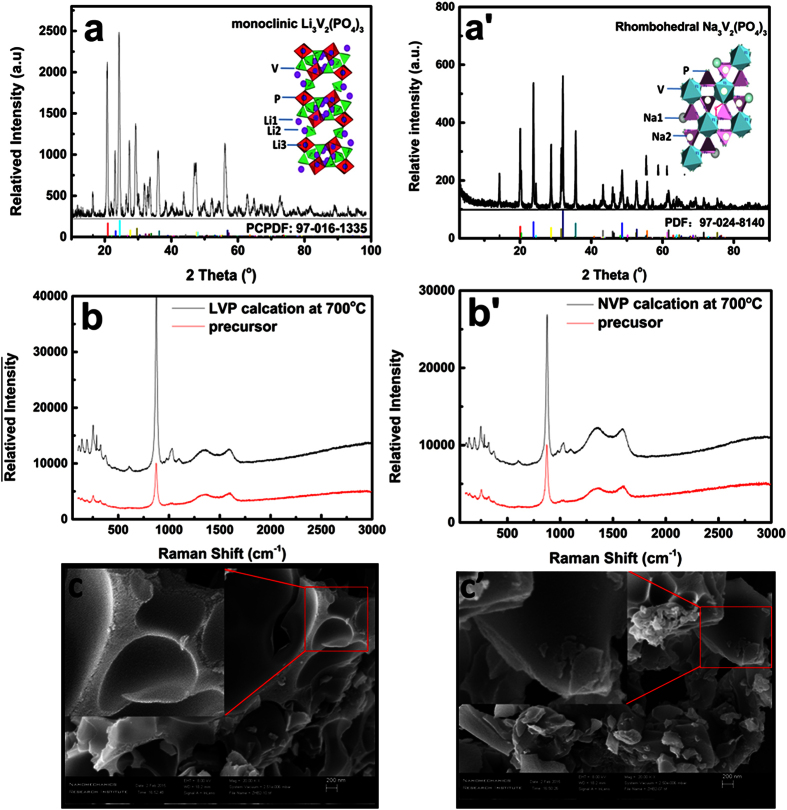
XRD diffraction patterns, Raman spectrum and SEM images of LVP (**a–c**) and NVP (**a′–c′**), the inserts of c and c′ is the low amplification images.

The physical characters and phase purity of as synthesized MVP-C composite were evaluated. The monoclinic Li_3_V_2_(PO_4_)_3_ with the space group P2_1/n_ (PCPDF CARD:97-016-1335) can be defined from the XRD pattern of LVP-C composite (Fig. 2a). Three main peaks at 2θ = 20.6°, 24.2° and 27.8° are very sharp, which means good crystalline degree with the initial lattice size of about 21 nm calculated by Scherrer equation. The XRD pattern of NVP-C (Fig. 2a′) is also indexed as typical rhombohedral phase with the space group R-3c (PCPDF CARD:97-024-8140). No secondary phase is detected on the XRD patterns, which indicates that carbon in these two composites exist in an amorphous state with low content. Raman spectrum (Fig. 2b,b′) confirms that the C_sp2_ hybrid is dominant compared with C_sp3_ for the large I_G_/I_D_ value, therefore electronic conductivity is sufficient for high rate application. SEM images of the MVP-C composites obtained by sol-gel method are shown in Fig. 2c,c′. These two black MVP-C powders are bulk with uniform flake-like morphologies and the wall thickness is around 100 nm. ICP-AES analysis reveals that the content of LVP and NVP in the composites is about 91.44 wt% and 87.29%, respectively. The specific capacity is calculated according to the total weight of the composites.

**Figure 3:**
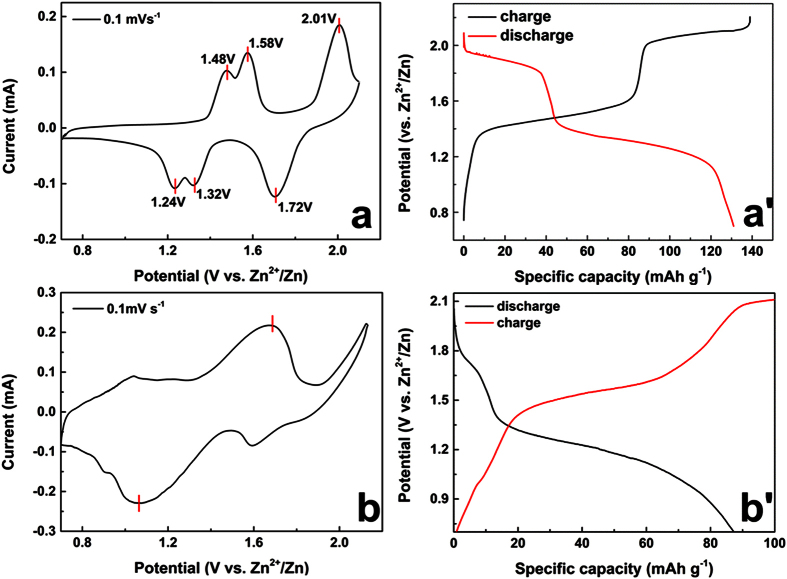
Stabled CV curves of MVP cathodes (**a**: LVP, **b**: NVP) at the scan speed of 0.1 mV s^−1^; the charge/discharge profile of MVP//Zn batteries (a′: LVP, b′: NVP) in 1M Li_2_SO_4_ + 2M ZnSO_4_ (pH = 4.0) in the electrochemical window from 0.7 to 2.1 V at 0.2C.

Electrochemical mechanism of MVP//Zn ReHABs in 1M Li_2_SO_4_ + 2M ZnSO_4_ were further studied by CV technique and charge/discharge. For the CV curve of LVP//Zn battery (Fig. 3a), there are three pairs of positive/negative peaks in the range from 0.7 V to 2.1 V, suggesting that the Li ions are partially mobile during the charge-discharge process. The two positive peaks around 1.48 and 1.58 V are corresponding to the extraction of the first Li ion, since there is an ordered Li_2.5_V_2_(PO_4_)_3_ phase according to its behaviour in organic electrolyte system. Good reversibility was observed by comparing the two pairs of redox peaks at around 1.58 V/1.32 V and 1.48 V/1.24 V, respectively. The third pair of redox peaks at around 1.72 V/2.01 V corresponds to the phase transition between Li_2_V_2_(PO_4_)_3_ and LiV_2_(PO_4_)_3_^16^ which relates to the extraction/insertion of the second lithium ion. These peak potentials are lower than that of the OER, indicating a stable electrochemical performance. However, the third lithium ion in LVP lattice is difficult to be pulled out from the lattice because a serious OER of H_2_O occurred previously when charge/discharge voltage window is higher to 2.2 V vs. Zn^2+^/Zn (Fig. 3a,a′). Nevertheless, extraction of two lithium ions from LVP structure is possible in present ReHABs environment, which results to a high theoretical specific capacity of 131 mAh g^−1^. This fact is encouraging for conducting further investments of LVP//Zn in the future. The plateaus of charge/discharge profile (Fig. 3a′) demonstrated that LVP//Zn has a two-lithium-ion insertion/ extraction behaviour.

Compared with the LVP//Zn, the stabled CV curves of NVP//Zn battery (Fig. 3b) in 1M Li_2_SO_4_ + 2M ZnSO_4_ showed only one pair of oxidation/reduction flat peaks around 1.55 V/1.35 V after dozens cycles. Its charge/discharge profile (Fig. 3b′) also confirmed that the NVP//Zn has one pair of obvious charge/discharge plateaus. This result is in good agreement with that of its behaviour in organic electrolyte^39^,^40^. Because of the complexity of Li^+^ and Na^+^ mixed aqueous electrolyte, the electrochemical behaviour of NVP cathode exhibits remarkable difference between initial cycles and the following cycles. Its electrochemical mechanism will be discussed later.

Usually the electrochemical reactions in cathodes mainly depend on their insertion/extraction of Li^+^/Na^+^ ions in the lattice of materials, charging/discharging depth and the reversible change of crystal structure. Similar to organic system, there is a complicated multistep charge/discharge process will occur between LVP and its intermediates (Li_3−x_ V_2_(PO_4_)_3_, 0 < x < 3). in aqueous electrolyte (Eq. 1). Different to LIBs in organic electrolytes, the anode reaction is related to the zinc ion precipitation/dissolution on the surface of anode collector and its half cell reaction is shown in Eq. 2. The redox reactions during charge/discharge processes of LVP-C//Zn ReHABs can be suggested as following:


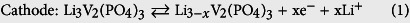



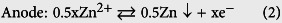


Full battery reaction:


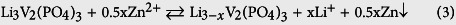


**Figure 4:**
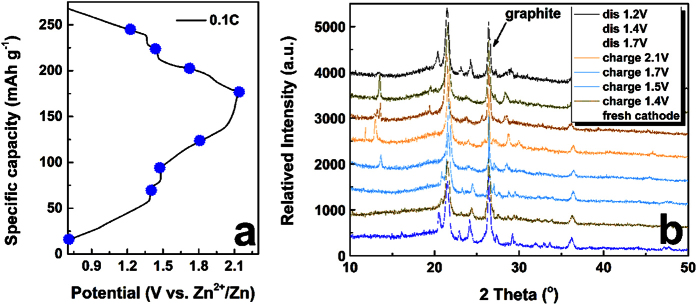
(**a**) Initial charge/discharge profile at 0.1C; (**b**) the corresponding ex-XRD patterns of LVP cathode during charge/discharge process.

The electrochemical mechanism and structure transitions of LVP cathode during charge/discharge processes were explored by *ex-situ* XRD patterns as presented in (Fig. 4a,b). Obvious diffraction peak shifts and appearance or disappearance of new peaks are observed in the charge/discharge process. Although the ionic radius of Zn^2+^ is similar to Li^+^, its charge is +2 and the reversible charge/discharge with minor change of XRD patterns demonstrated that it can not intercalate into the lattice of LVP easily. No new phase which is related to zinc created confirmed the structure stability of LVP in aqueous electrolyte. After one charge/discharge cycle, the LVP diffraction peaks recovered totally, confirming good electrochemical reversibility.

Further, the electrochemical mechanism was summarized as follows: The two positive peaks around 1.58 V and 1.48 V correspond to the extraction of the first Li ion in two steps (Eq. 4 and Eq. 5), since there is an ordered Li_2.5_V_2_(PO_4_)_3_ phase according to its behavior in organic electrolyte system. Very perfect reversibility is observed compared the two pairs of charge/discharge peaks at 1.58 V/1.32 V and 1.48 V/1.24 V, respectively.


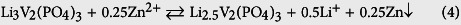



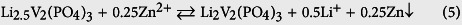



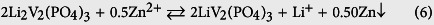


Another pair of reversible charge/discharge plateaus located at about 2.01 V/1.72 V are related to the insertion/extraction of the second Li^+^ and its electrochemical reaction is described as Eq. 6. The potential difference (ΔV_p_) of this pair of redox peak is about 0.29 V, indicating little polarization occurred compared with the first Li^+^ insertion/extraction process. Although theoretically the third Li ion can be delithiated at high voltage about 2.4 V, serious decomposition of hydrogen oxide leaded to the destruction of electrolyte. Based on the former exploratory experiment, we make sure the possibility for these electrochemical ReHABs in the voltage window between 0.7 V to 2.2 V.

**Figure 5:**
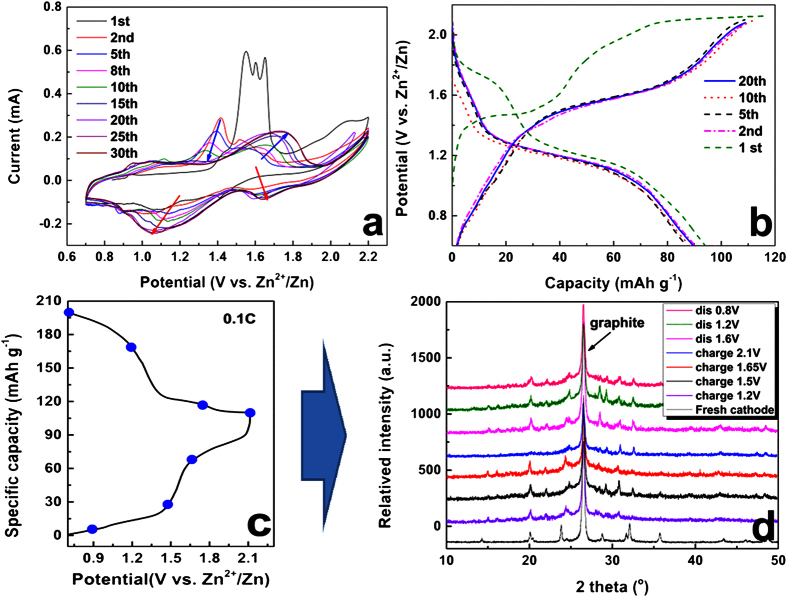
(**a)** The CV curves of NVP cathode in different cycle with scan speed of 0.1 mV s^−1^; (**b**) The first 20 cycles of charge/discharge of NVP/Zn battery at 0.2C; (**c,d**) Initial charge/discharge profile at 0.1C, and the corresponding ex-XRD patterns of NVP cathode during charge/discharge process.

As for the NVP cathode, the electrochemical mechanism is more complicated due to its co-insertion/extraction of Na^+^ and Li^+^. To further study its electrochemical behaviour in mixing ions electrolytes, we explored its CV (Fig. 5a) and charge/discharge performance (Fig. 5b) in aqueous electrolytes with Li^+^ and Zn^2+^ ions.

Totally, the electrochemical reactions on NVP electrodes are speculated as follows:

Initial charge process:


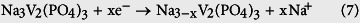


Following charge/discharge process:


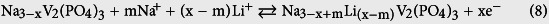


CV curves presented in Fig. 5a strongly supported this speculation. The initial cycle of NVP//Zn battery in Li-containing aqueous electrolyte exhibits obviously different to that of the following ones (Fig. 5a). The initial oxidative peaks around 1.6 V, which is related to the extraction of Na^+^ ion (Eq. 7), disappeared at the second cycle, indicating an entirely different extraction mechanism after Na^+^ were extracted. Two new oxidative peaks around 1.4 V and 1.5 V appeared. However, these two peaks also gradually disappeared (Fig. 5) and one new oxidative peak became gradually stronger and positively shifts to 1.70 V. The reduction peaks around 1.2 V shifted negatively and turn stable after 25^th^ CV cycle. Another reduction peak around 1.65 V gradually appeared and gets stable after 25 cycles. The integral area of positive/negative peaks, which represents one style of capacity, increases gradually to be stable, especially on the negative peak area.

Therefore, we speculated that a new balance with stabled structure of 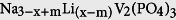 formed after dozens cycles of charge/discharge, and the M^+^ ions insertion/extraction became reversible and stable, as is shown in Eq. 8. Because of the low concentration of Na^+^ in the electrolyte, the followed extraction/insertion process should be mainly related to Li^+^ ion.

The charge/discharge profiles of NVP//Zn are also consistent to that of CV test. As shown in (Fig. 5b), the first charge curve with one plateaus around 1.5 V is attributed to the extraction of Na^+^ from NVP (Eq. 7). However, the following several charge/discharge process showed that charge plateaus around 1.5 V slowly moved to 1.6 V, confirming a different extraction energy with that of initial one. Similarly, the discharge plateaus at 1.75 V disappeared, and a longer plateau around 1.2 V became more obvious, confirming the energy and structure change of NVP during the discharge process. A stable charge/discharge profile with discharge capacity of 95 mAh g^−1^ was obtained after 20 cycles.

Ex-XRD (Fig. 5d) result supported this speculation when detecting the phase and structure change of NVP cathode in the charge/discharge process. The fresh cathode is indexed to be typical rhombohedral Na_3_V_2_(PO_4_)_3_ and current collector graphite (002). With the charge depth increases from 0.8 V to 2.1 V, more sodium ions were extracted from the lattice of NVP, and the initial rhombohedral phase Na_3_V_2_(PO_4_)_3_ disappeared, companying with other obvious different diffraction peaks appeared, which should be attributed to that of intermediates 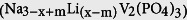. These intermediates were also not indexed as NVP or LVP. The followed discharge process was complicated because of the insertion of Li^+^ ions and Na^+^ ions. This has also been demonstrated by other literatures about sodium-ions batteries in organic electrolytes, in which Na^+^ can be inserted/extracted reversibly on NVP^34^,^38^. The final full discharged cathode could not be assigned as the original rhombohedral Na_3_V_2_(PO_4_)_3_. This result confirmed that NVP has obvious different charge/discharge mechanism compared with LVP. High concentration of Li^+^ ion compared with very low concentration of Na^+^ ion leaded to a new balance, and the insertion/extraction of Li^+^ ion has main contribution to the total capacity.

Besides of the structure stability of MVP cathodes, the electrochemical performance of the designed MVP//Zn ReHABs enormously depends on other factors, including the pH and concentration of aqueous electrolytes, the Zn dendrite formation, and the self-discharge of batteries.

**Figure 6:**
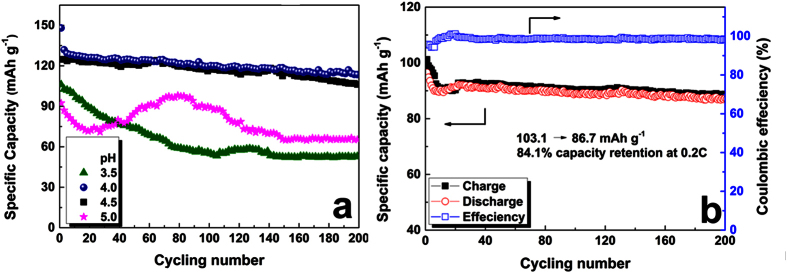
(**a**) The cycling performance of LVP//Zn battery in the aqueous electrolytes with different pH of 2.5, 4.0, 4.5 and 5.0 at 0.2C; (**b**) Cycling performance of the NVP//Zn ReHABs with pH = 4.0 at 0.2C.

The effect of electrolyte’s pH and concentration on the stability and cycling performance of LVP in aqueous electrolyte was evaluated detailedly (Fig. 6a). The electrolyte concentration is a very important factor which affect the performance of batteries. First, a balance of Li^+^ and Zn^2+^ should be considered, therefore Li^+^:Zn^2+^ of 1:2 is a good ratio. Although the lower concentration of Li^+^ or Zn^2+^ is helpful for fast migration speed, too low concentration is not enough for our batteries because the cathode layer thickness is about 100 μm with active material loading high up to 6 mg/cm^2^. Additionally, the challenge of oxidation and corrosion of graphite paper(current collector) also requires less volume of electrolyte. Therefore, in this work, we used 1M Li_2_SO_4_ + 2M ZnSO_4_ as electrolyte. Moreover, other works about dendrite and surface species of Zn have been studied in our recent accepted paper^41^.

The effect of pH on the electrochemical performance were evaluated and an optimized pH about 4 to 5 is more stable for our system. Considering that the Zn^2+^ ion will deposit in neutral and alkaline mediates, we used dilute LiOH or H_2_SO_4_ to modify aqueous electrolyte to different pH from 3.5 to 5 to evaluate its cycling performance. The discharge capacity of the LVP//Zn batteries in low pH electrolyte (pH = 3.5) descends fast because of the slow dissolution of Zn anode. When the pH increases to 5.5, the OH^−^ concentration in electrolyte increases and Zn^2+^ will precipitate and lost its mobility slowly. Moreover, the decomposition of water caused by polarization will lead to the increase of salt concentration, even saturated and precipitated. Therefore, the ReHABs will not work well in inadaptable pH electrolytes.

Optimistically, a modest pH range of 4–5 is much better than others for the stable cycling performance. As shown in Fig. 6a, the LVP//Zn battery with pH = 4 and 5 shows more stable cycling performance after 200 cycles with the discharge capacity of 113.5 mAh g^−1^ and 106.2 mAh g^−1^, respectively. While LVP//Zn batteries with pH = 3.5 and 5 exhibit worse cycling performance because of serious dissolution of Zn anode or precipitation of electrolyte. LVP//Zn battery with pH = 4 exhibits the highest capacity retention ratio of 85.4% after 200 cycles at 0.2 C. The coulombic efficiency increased from 86.9% to 97%, indicating gradually improved kinetic performance after several charge/discharge activation cycles. Therefore, a modest pH of 4 is adaptable for this hybrid aqueous electrolyte battery with Zn as anode.

For the NVP//Zn battery operating in Li-containing electrolyte (pH = 4.0), the initial cycles shows capacity descend like that of LVP because of structure change in the charge/discharge process. The transformation of Li-containing NVP to the complex Na_3−x+m_Li_(x−m)_V_2_(PO_4_)_3_ that lead to the shift of charge/discharge plateaus, has been observed in Fig. 5a,b. The low concentration of extracted Na^+^ ion in aqueous electrolyte cause to low insertion level of Na^+^ ion in NVP, and a competition and balance of Na-Li co-insertion/ extraction of NVP with gradually stabled specific capacity. In the initial several cycles, more sodium ion were extracted, and more lithium ions were inserted into NVP. This transitional charge/discharge behavior became to stable after 30 cycles. This result is consistent to the change tendency of CV curves (Fig. 5a). Therefore, a stable cycling performance of NVP//Zn battery (Fig. 6b) is remained in aqueous electrolyte of pH = 4 with discharge capacity retention ratio of 84.1% (86.7 mAh g^−1^) at 0.2C, and the coulombic efficiency was kept to 98.1% after 200 cycles charge/discharge.

**Figure 7:**
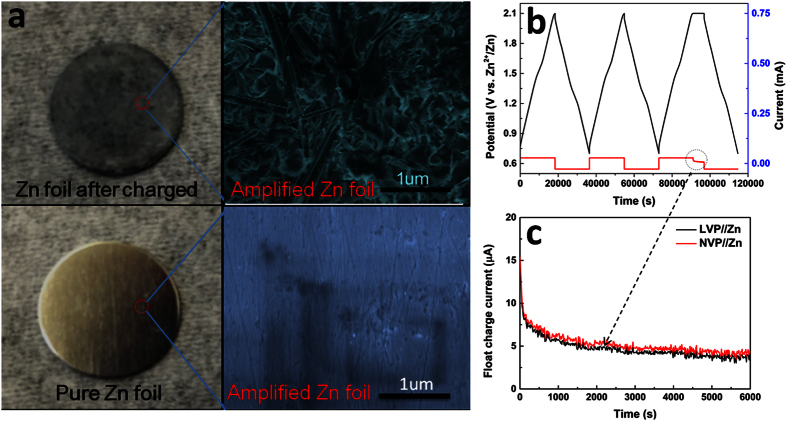
(**a**) The surface morphology of Zn anode of LVP/Zn battery before and after 20^th^ charge/discharge cycles; (**b**) the charge/discharge of LVP//Zn and float charge current test; (**c**) the corresponding amplified profile of float charge current.

The float charge current test, which is one important factor to evaluate the stability and self-discharge behavior of batteries, were carried out after galvanostatically charged at 0.2C to 2.1 V, followed by charging at constant voltage of 2.1 V for 6000 s. Smaller float charge current represents less self-discharge coming from Zn dendrite or unstable of electrode materials. As shown in Fig. 7b, the float charge currents of these two batteries dropped quickly to less than 6 μA cm^−2^, and became stable. This means little self-discharge because of dissolution of Zn, corrosion of collector, decomposition of electrolyte, or structure destroy of electrode. Therefore, good electrochemical stability of LVP//Zn and NVP//Zn were obtained, which is similar to other work we recently reported^41^.

The formation of Zn dendrite during discharge process is another important factor which affects the stability and cycling performance of ReHABs. It has been reported by other aqueous electrolyte batteries with Zn as anode that Zn dendrite will cause serious inner shortcut if Zn dendrite grows, punctures separator and contacts with cathode. In our MVP/Zn batteries, the Zn dendrite also formed when discharging it. As shown in Fig. 7a, after discharging for 20 cycles, the smooth and metallic luster surface of Zn anode became dark grey and rough. In high magnification SEM image, the Zn dendrites with around 2 μm in length were observed as well rough surface, which has potentially danger to the lifetime of battery when the dendrites grow longer enough to contact with cathode. Optimistically, more works will be done to prevent the formation of Zn dendrite in the future, such as using gel electrolyte or additives, coating by other materials.

**Figure 8:**
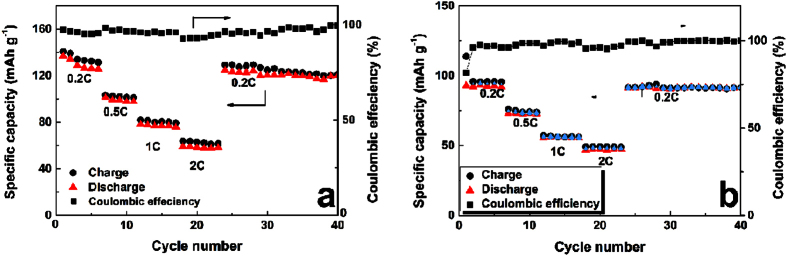
C-rate profiles of LVP//Zn (**a**) and NVP/Zn (**b**) ReHABs in 2M Li_2_SO_4_ + 1M ZnSO_4_ aqueous electrolyte with pH = 4.

Rate capability of MVP//Zn battery is presented in Fig. 8. Totally, the charge and discharge capacities of these two batteries decrease companying with the increased C-rate. For LVP//Zn battery, the cell still exhibits a discharge capacity of 62 mAh g^−1^ at a high C-rate of 2 C. When the C-rate comes back to 0.2 C, a specific capacity of 118.3 mAh g^−1^ still was obtained, confirming the robustness of the battery. Similarly, NVP/Zn battery has good C-rate performance of 51 mAh g^−1^ at a high C-rate of 2 C. The main reason should be attributed to their two-level structure in morphology and the fast Li^+^ and electron transfer in MVP-C composite, as well as high ion conductivity of aqueous electrolyte. However, the normalized energy density of LVP//Zn and NVP//Zn is 36.3 Wh kg^−1^ and 25.1 Wh kg^−1^, respectively. This value is the same level of the commercial Pb-acid battery, indicating very large potential to improve their electrochemical performance in future. The low medium voltage of LVP//Zn (1.23 V) and NVP//Zn (1.11 V) caused by electrode polarization is one important factor that affect the energy density. More works should be focused on improve their electrode structure, optimize separator and electrolytes.

In conclusion, we reported a new rechargeable hybrid aqueous battery with NASICON-type M_3_V_2_(PO_4_)_3_ (M = Li, Na) and zinc as the cathode and the anode, respectively. Totally, these two phosphate-based cathodes exhibit different electrochemical behaviour because of their inherent structure. A binary ions-containing (Zn^2+^, Li^+^/Na^+^) electrochemical reaction existed with a reversible charge/discharge capacity of 113.5 mAh g^−1^ at 0.2 C on LVP//Zn battery after 200 cycles. Electrochemical mechanism study shows that LVP is stable in aqueous electrolyte within 0.7 V to 2.1 V and two lithium ions can be reversibly delithiated. In the case of NVP//Zn battery, owing to the complicated two-ions mixed insertion/extraction mechanism, the stabled charge/discharge profiles were obtained after 25 cycles with specific capacity of 86.7 mAh g^−1^ at 0.2 C after 200 cycles. The development of this ReHABs with MVP (M = Na, Li) as cathodes may have a significant impact on electrochemical energy storages owing to its low cost and safety. More work should be focused on improving the cycling performance, optimizing electrolyte and counter electrode, well as the dissolution and dendrites formation on the Zn anode in weak acidic electrolytes.

## Methods

### Synthesis of M_3_V_2_(PO_4_)_3_ (M = Li, Na)

The MVP-C composite was synthesized by typical sol-gel method.^13^ 2.0000 g of commercial V_2_O_5_, lithium carbonate (or sodium carbonate), NH_4_H_2_PO_4_ powders and citric acid (molar ratio of 1:3:2:3) were mixed, then 10 mL of deionized water-ethanol (V:V = 1:1) was added. The mixture was stirred and heated at 80 °C to yield a dried gel. The gel was treated at 350 °C for 8 h in N_2_ atmosphere. Finally, the powders were tableted and treated at 700 °C for 8 h in N_2_ atmosphere. The obtained powder is named LVP-C and NVP-C, respectively.

### Characterization

The X-ray diffraction (XRD) patterns of the samples were recorded on a Rigaku D/max 2200 X-ray diffractometer with Cu Ka radiation (λ = 1.54056 Å). Morphology analysis was performed with a JEOL JSM−6700F field emission scanning electron microscope (FESEM). Raman spectrum was carried out on a laser Raman spectrometer (Renishaw inVia plus) with a laser wavelength of 633 nm.

### Cells assemble and electrochemical measurements

The MVP cathode was fabricated by coating a mixture of MVP powder, acetylene black, and 10 wt% PVDF in NMP (weight ratio of 8:1:1) on graphite paper. After drying at 80 °C for 12 hours, the cathode was cut as disk with diameter of 9 mm. Swagelok-type cells were assembled from Zn foil as anode and MVP-C as cathode, separated by a glass fibre separator containing aqueous electrolyte (1M Li_2_SO_4_ + 2M ZnSO_4_). The pH of electrolyte was modified by dilute LiOH or H_2_SO_4_ with the assistant of pH meter. The cells were charge-discharge galvanostatically between 0.7 and 2.1 V vs. Zn^2+^/Zn for the cyclability and C-rate tests on CT2001A Land Battery Testing System. The float charge current test was carried out by galvanostatically charge to 2.1 V, followed with constant voltage charge at 2.1 V to test the change of current. Electrochemical reaction mechanism was investigated by cyclic voltammetry (CV) on an electrochemical workstation (CHI 660D) and *ex-situ* powder X-ray diffraction (XRD).

## Additional Information

**How to cite this article**: Zhao, H. B. *et al*. Novel Rechargeable M_3_V_2_(PO_4_)_3_//Zinc (M= Li, Na) Hybrid Aqueous Batteries with Excellent Cycling Performance. *Sci. Rep*. **6**, 25809; doi: 10.1038/srep25809 (2016).
